# 3D‐printed headrest for frameless Gamma Knife radiosurgery: Design and validation

**DOI:** 10.1002/acm2.12956

**Published:** 2020-06-30

**Authors:** Garrett C. Baltz, Tina Briere, Dershan Luo, Rebecca M. Howell, Shane Krafft, Eun Young Han

**Affiliations:** ^1^ Department of Radiation Physics The University of Texas MD Anderson Cancer Center Houston TX USA

**Keywords:** 3D printing, Gamma Knife, immobilization, radiosurgery

## Abstract

**Purpose:**

Frameless Gamma Knife stereotactic radiosurgery (SRS) uses a moldable headrest with a thermoplastic mask for patient immobilization. An efficacious headrest is time consuming and difficult to fabricate due to the expertise required to mold the headrest within machine geometrical limitations. The purpose of this study was to design and validate a three‐dimensional (3D)‐printed headrest for frameless Gamma Knife SRS that can overcome these difficulties.

**Materials and methods:**

A headrest 3D model designed to fit within the frameless adapter was 3D printed. Dosimetric properties of the 3D‐printed headrest and a standard‐of‐care moldable headrest were compared by delivering a Gamma Knife treatment to an anthropomorphic head phantom fitted with an ionization chamber and radiochromic film. Ionization measurements were compared to assess headrest attenuation and a gamma index was calculated to compare the film dose distributions. A volunteer study was conducted to assess the immobilization efficacy of the 3D‐printed headrest compared to the moldable headrest. Five volunteers had their head motion tracked by a surface tracking system while immobilized in each headrest for 20 min. The recorded motion data were used to calculate the average volunteer movement and a paired t‐test was performed.

**Results:**

The ionization chamber readings were within 0.55% for the 3D‐printed and moldable headrests, and the calculated gamma index showed 98.6% of points within dose difference of 2% and 2 mm distance to agreement for the film measurement. These results demonstrate that the headrests were dosimetrically equivalent within the experimental uncertainties. Average motion (±standard deviation) of the volunteers while immobilized was 1.41 ± 0.43 mm and 1.36 ± 0.51 mm for the 3D‐printed and moldable headrests, respectively. The average observed volunteer motion between headrests was not statistically different, based on a *P*‐value of 0.466.

**Conclusions:**

We designed and validated a 3D‐printed headrest for immobilizing patients undergoing frameless Gamma Knife SRS.

## 1 INTRODUCTION

The Gamma Knife Icon (Elekta, Stockholm, Sweden) system equipped with an integrated cone‐beam computed tomography (CBCT) allows for frameless stereotactic radiosurgery (SRS) treatments to be performed. Whereas traditional Gamma Knife treatments are delivered in one fraction with the patient immobilized using a stereotactic frame affixed to the patient's skull, frameless treatments are delivered with the patient immobilized in a moldable patient‐specific headrest and a thermoplastic mask. This system enables fractionated Gamma Knife treatments. A CBCT of the daily setup, which is used to correct for daily patient positioning, is acquired and registered to the planning CT or MRI. Intrafraction motion is monitored using the high definition motion management (HDMM) system, which uses an infrared (IR) camera to monitor an IR reflective marker on the patient's nose as a surrogate for head motion.^1^ The combination of CBCT positioning and HDMM has been demonstrated to provide sufficient localization and motion management for Gamma Knife SRS treatments.^2^, ^3^, ^4^

The patient‐specific mask and headrest used for immobilization are created during the patient's treatment simulation on the Gamma Knife unit. Creation of the headrest during simulation can be challenging due to a combination of factors, especially for centers with limited experience in creating radiotherapy immobilization, such as Gamma Knife units in neurosurgery departments, or for centers that frequently rotate staff who may have limited previous experience with Gamma Knife simulations. The primary challenge is being able to mold the patient headrest within the geometrical limitations of the Gamma Knife imaging and treatment system. The CBCT system has a limited field of view, making it critical that the patient is indexed in a location within the frameless adapter such that the entire skull can be imaged for accurate registration. Treatment collisions also represent a challenge, as target locations very anterior or inferior can potentially be untreatable due to possible collision of the patient and the source assembly. Commercial moldable headrests currently used are activated by heat or water and have a limited amount of time to be shaped, which can make it challenging to position the patient in an optimal treatment position considering the aforementioned geometrical limitations. This problem has been previously described in the literature by Li et al. who noted that the creation of the immobilization devices is challenging due to time limitations and the experience required to create reproducible and effective immobilization.^4^ These challenges, as well as the possibility that a poorly fabricated headrest could negatively affect a patient's treatment, motivated the research of an alternative solution to fabricate the headrest for frameless Gamma Knife immobilization.

A standardized 3D‐printed headrest could alleviate the challenges associated with creating the headrest during simulation. A standardized headrest model based on previous examples of good headrests and known good patient indexing could minimize the potential of imaging and collisional issues. This standardized headrest would lower the reliance on user experience and patient cooperation required to create a good headrest ad hoc during the simulation, as well as reduce variability of immobilization between patients.

Three‐dimensional printing has been previously demonstrated in patient immobilization applications for radiotherapy. Pham et al. used 3D‐printed head models from patients' CT scans to fabricate thermoplastic masks before a patient's simulation.^5^ Haefer et al. and Sato et al. demonstrated the feasibility of using 3D‐printed masks for immobilization in linac‐based radiotherapy of head and neck cancers.^6^, ^7^ The purpose of this study was to design and validate a 3D‐printed headrest for patient immobilization for frameless Gamma Knife SRS treatments.

## 2 MATERIALS AND METHODS

### 2.A Standardized headrest CAD design and fabrication

**FIG. 1:**
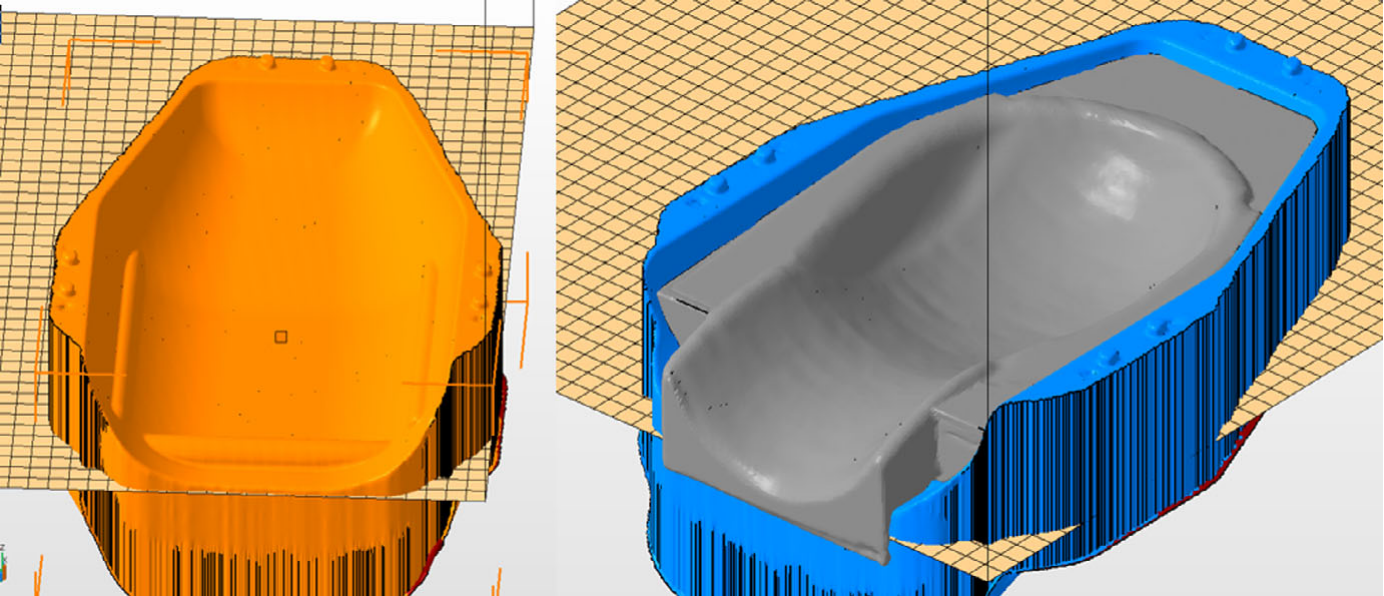
Left: three‐dimensional (3D)‐model of frameless adapter acquired with optical scan. Right: 3D‐model of headrest (grey) in frameless adapter (blue).

In frameless Gamma Knife treatments, the patient is immobilized for treatment within the frameless adapter affixed in the patient fixation mount on the treatment couch. The adapter provides support for the headrest and has locking points for a thermoplastic mask. The bottom of the adapter has three ridges that are used for indexing the location of the headrest. A 3D model capturing the geometry of the frameless adapter was generated by acquiring an optical scan of it using a David SLS‐3S 3D Scanner (HP Inc., Palo Alto, CA). This 3D model was imported into Netfabb CAD software (Autodesk, San Rafael, CA) and used as a form to shape the bottom of the headrest, as shown in Fig. 1. This ensured the bottom of the headrest would interlock with the indexing and fit securely in the adapter. Models of an anthropomorphic head phantom and a headrest used for linac‐based SRS were imported from a CT scan and used to generate the shape of the headrest. The headrest model was designed to be as thin as possible and to place the head optimally within the CBCT field of view.

The headrest was 3D printed in polylactic acid (PLA) material using a GigaBot 3 3D printer (re:3D Inc., Houston, TX). The printer settings used were a nozzle temperature of 240°C and a bed temperature of 60°C. The model was printed with a layer height of 200 microns, two shell layers, and 2% infill percentage with a rectilinear pattern, leaving the headrest mostly hollow yet strong enough to support a patient's head.

### 2.B Dosimetric study

Treatment planning for Gamma Knife is done in Leksell GammaPlan software (Elekta Instrument AB, Stockholm, Sweden). In typical clinical use, dose calculations are performed using the TMR algorithm, which calculates dose based on homogeneous water within the shape of a patient's skull as defined by the planning image data set.^8^ As no heterogeneity corrections are performed, the headrest is not modeled when generating the treatment plan. This meant it was necessary to conduct a dosimetry study to determine if the 3D‐printed headrest achieves a dosimetrically equivalent treatment compared to the conventional moldable headrest used.

First, the radiological properties of the 3D‐printed headrest were compared to the standard‐of‐care moldable headrest. A CT scan of the 3D‐printed headrest described in Section II.A. and that of a Moldcare Cushion (Qfix, Avondale, PA) representing the standard of care were acquired with a Philips Big Bore CT Scanner (Philips Healthcare, Andover, MA, USA) using settings of 120 kVp, 400 mAs, and slice thickness of 1 mm. The CT scan was imported into 3D‐Slicer,^9^ where the 5 central slices of each headrest were segmented and the average HU and standard deviation were recorded.

Further dosimetric study compared the headrests in the context of a frameless Gamma Knife treatment of a head phantom and consisted of a point dose measurement with an ion chamber and a dose distribution measurement with film.

#### 2.B.1 Ion chamber

**FIG. 2:**
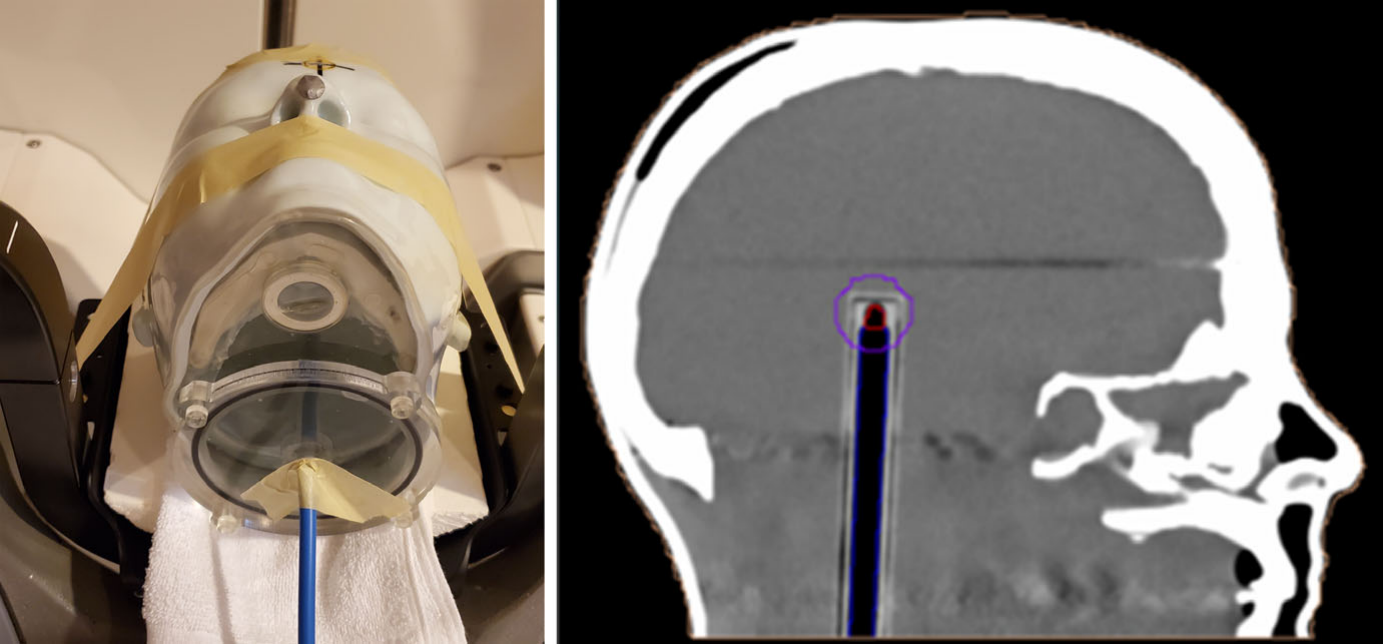
Left: RTSafe Phantom in standardized three‐dimensional‐printed headrest with ion chamber in place. Right: Rendering from GammaPlan showing location of ion chamber and volume treated.

The ion chamber measurements were performed using an RTSafe anthropomorphic SRS head phantom (PseudoPatient, RTsafe, Artotinis, Greece), fitted with a PTW TN31010 0.125cc ionization chamber in the center of the cranium. A CT scan of the head phantom was acquired with a Philips Big Bore CT scanner and imported into GammaPlan. A treatment plan was generated with a single 16 mm shot centered on the chamber volume with a prescription of 2 Gy to the 47% isodose line, which corresponded to an average dose of 4.22 Gy to the chamber active volume. The phantom was set up in the Gamma Knife frameless adapter in the standardized 3D‐printed headrest and secured with masking tape. The experimental setup and treatment plan used are shown in Fig. 2.

The planned treatment was delivered to the phantom and the ionization chamber integrated charge was recorded. Two repeat ionization measurements were acquired for this setup. The procedure was repeated with the head phantom setup in a Moldcare Cushion that had been formed to the phantom, which represents the current standard‐of‐care headrest.

#### 2.B.2 Film

**FIG. 3:**
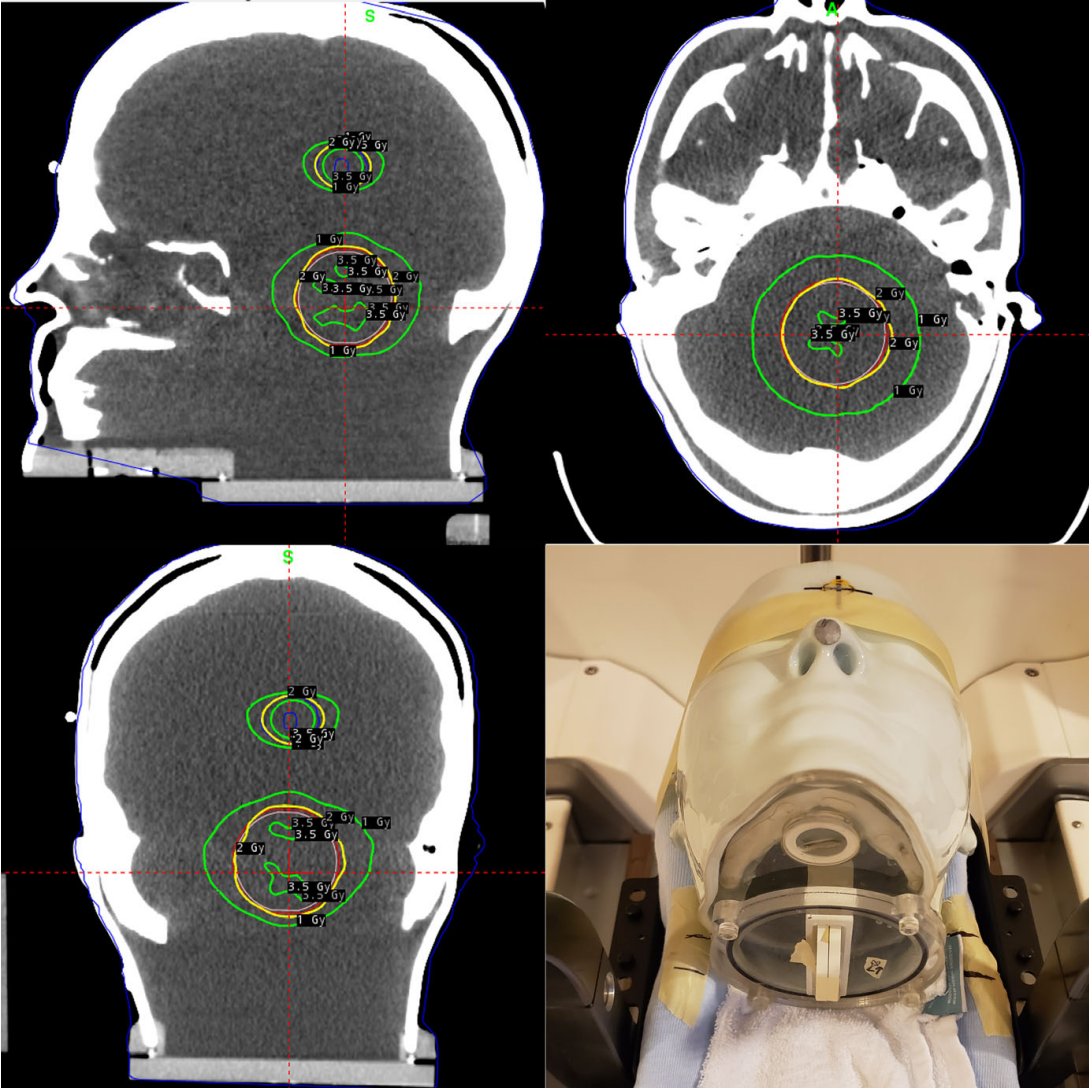
Top and Bottom Left: Renderings of treatment plan used for film measurement. Bottom Right: Experimental setup used for anthropomorphic head phantom film measurement for standard‐of‐care headrest.

A second set of irradiations was performed using the same experimental setup, with radiochromic EBT3 film (Radiation Products Design Inc., Albertville, MN) inserted into the phantom in place of the ionization chamber. A Gamma Knife treatment plan was generated in GammaPlan to deliver 2 Gy to the 50% isodose line to both a 4‐cm‐diameter PTV and a 2‐cm‐diameter PTV, shown in Fig. 3. The planned treatment was delivered to the head phantom, once with the phantom setup with the standard‐of‐care cushion, and once with the phantom in the 3D‐printed headrest.

The irradiated films were scanned using a 10000XL flatbed scanner (Epson, Long Beach, CA). MyQA software version 2.9.23 (IBA Dosimetry, Schwarzenbruck, Germany) was used to calculate the Gamma Index^10^ with the standard‐of‐care film as reference and the 3D‐printed film as comparison using 2% dose difference, 2 mm distance to agreement, global normalization, and 20% dose threshold as the gamma index criteria.

### 2.C Immobilization study

Minimizing patient motion during treatment is the primary purpose of immobilization, and is particularly critical for SRS treatments that utilize smaller treatment margins than non‐SRS treatments. In order to be used clinically, a standardized 3D‐printed headrest must be able to immobilize the patient comparably to the current patient‐specific headrest used. A study was conducted to compare the immobilization efficacy of the standardized 3D‐printed headrest and the current patient‐specific standard‐of‐care headrest.

Intrafraction patient motion during frameless Gamma Knife treatments is monitored with the HDMM system. If the system measures patient movement in excess of a predefined threshold, typically on the order of 1.5 mm, the system will pause the treatment until the patient positioning is back within tolerance. This system could be used to provide an accurate *in vivo* measurement of the immobilization efficacy of the two headrests. However, leakage radiation is always present in the vicinity of the Gamma Knife, even when the shield doors are closed. In the interest of complying with ALARA principles, an immobilization study could not be conducted using the Gamma Knife built‐in HDMM system. Instead, AlignRT (VisionRT, London, UK), a commercial system used to perform surface‐guided radiation treatment was used to mimic the HDMM system. This system has been previously used to characterize new immobilization devices^11^, ^12^, ^13^ and provides the capability to monitor patient movement with submillimeter accuracy similar to the HDMM system on the Gamma Knife.

An immobilization study was conducted with 5 volunteers to compare movement while being immobilized in the 3D‐printed headrest vs while immobilized in a moldable standard‐of‐care headrest.

**FIG. 4:**
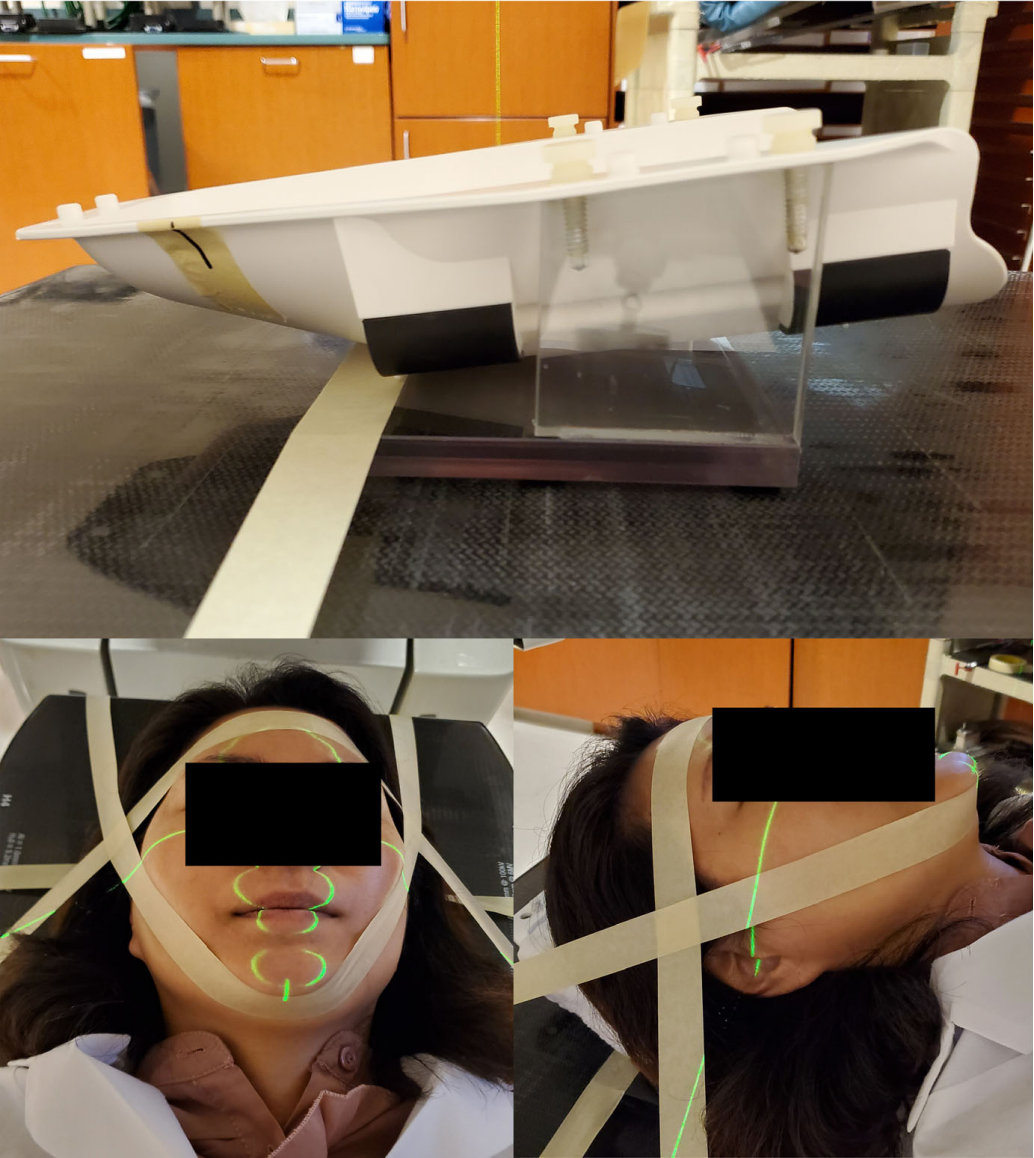
Top: Jig used to hold frameless adapter and headrest on linac treatment couch. Bottom: Images of tape immobilization used for volunteer study.

The experimental setup used for the immobilization study is presented in Fig. 4. A jig was made to hold the Gamma Knife MR frameless adapter on a linear accelerator treatment couch. The MR adapter has the same geometry as the adapter used for treatment.

Each volunteer had a custom standard‐of‐care headrest made by a radiation therapist with extensive experience making immobilization for frameless Gamma Knife treatments. The headrest was placed in the adapter, and the volunteer laid down. The volunteer's face was immobilized using two pieces of tape based on a low‐cost immobilization technique published by Rubinstein et al.^14^ This technique was chosen because it is a simple and robust immobilization technique that has been demonstrated to be able to provide sufficient immobilization for whole‐brain radiotherapy, and it was easier to reproduce and verify compared to making a new thermoplastic mask for each of the volunteer trials.

**FIG. 5:**
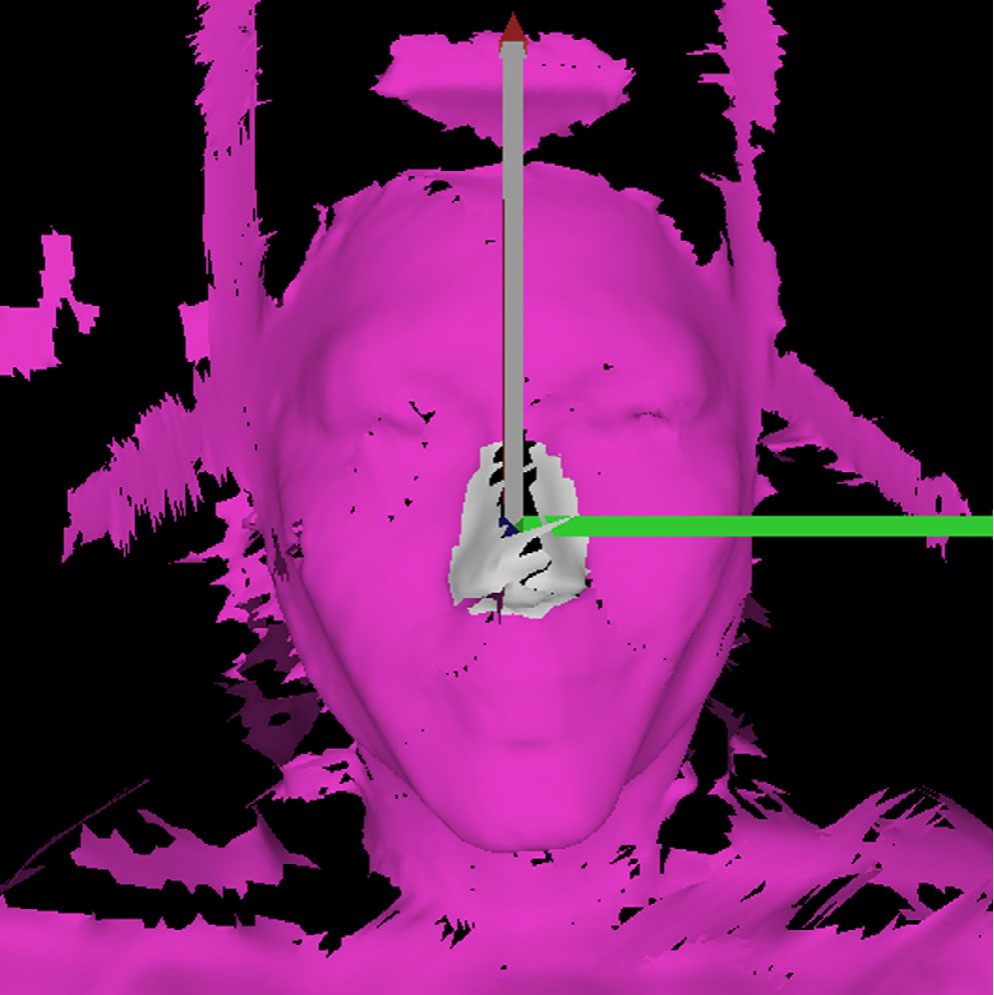
Rendering of VisionRT tracking software. Purple surface represents the reference scan acquired for the volunteer. Marked in white is the tracking ROI delineated to cover volunteer's nose.

Once immobilized in the headrest, a reference scan of the volunteer was acquired in AlignRT Version 5.1 and a region of interest (ROI) was delineated to cover the volunteer's nose, shown in Fig. 5. The nose was selected to mimic the motion observed by the HDMM system, which monitors an IR marker on a patient's nose tip. The volunteer was immobilized for 20 min and the vector distance from the reference position ($\sqrt{\Delta x^{2}+\Delta y^{2}+\Delta z^{2}}$) was recorded at a rate of approximately 6.5 Hz with the AlignRT system in the high‐resolution intracranial SRS mode. This procedure was first performed with the volunteer in the 3D‐printed headrest. After the 20‐min session, a 10‐min break was given and then the procedure was repeated with the volunteer in their respective standard‐of‐care cushion headrest.

The frame rate and accuracy of the motion tracking of the AlignRT system is dependent on the size of the surface ROI tracked. The uncertainty for motion tracking data acquired in this study was estimated by placing a stationary anthropomorphic head phantom in the headrest and tracking the ROI shown in Fig. 5 covering the phantom's nose for a minute.

The recorded vector distance vs time data for each volunteer was used to calculate an average displacement and standard deviation over the 20‐min session for each headrest. A paired two‐tailed *t*‐test was performed to determine if there was a significant difference in the average motion recorded for each volunteer between the 3D‐printed headrest and the standard‐of‐care headrest.

## 3 RESULTS

### 3.A 3D‐printed headrest

**FIG. 6:**
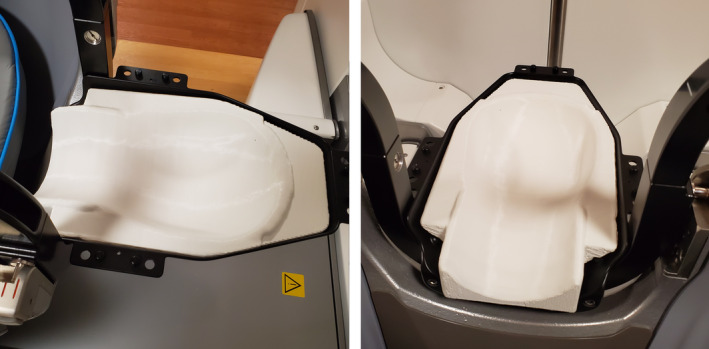
Standardized three‐dimensional‐printed headrest in Gamma Knife frameless adapter.

The headrest took 8 h to print and had a material cost of approximately $15. The final headrest fit tightly in the Gamma Knife frameless adapter, shown in Fig. 6.

### 3.B Dosimetric study

**FIG. 7:**
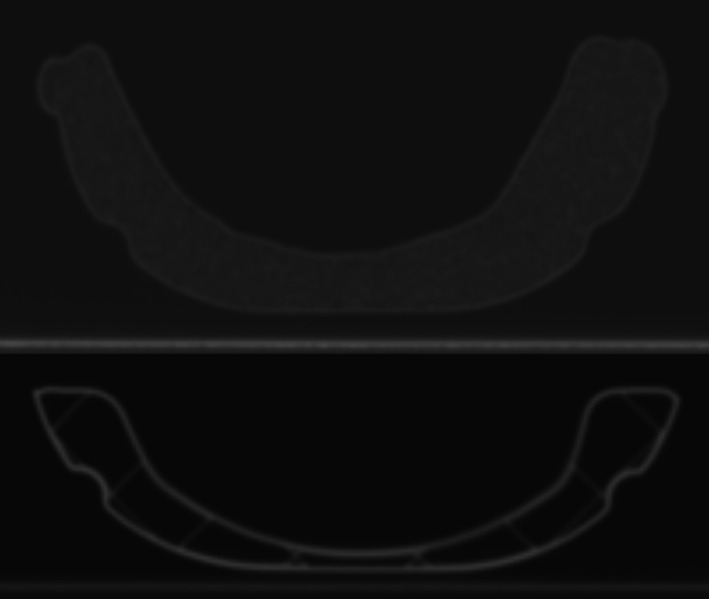
Top: CT scan of standard‐of‐care headrest. Bottom: CT scan of standardized three‐dimensional‐printed headrest.

Images from the CT scans acquired for the HU comparison of the headrests are shown in Fig. 7. The standard‐of‐care headrest had an average HU of −914 ± 31 corresponding to an effective density of 0.07 g/cm^3^, while the 3D‐printed headrest had an average HU of −848 ± 244 and effective density of 0.14 g/cm^3^. The inside of the 3D‐printed headrest is mostly air, which has a lower HU than the material in the standard‐of‐care headrest. However, the PLA material of the 3D‐printed headrest has a density of 1.10 g/cc, which leads to the 3D‐Printed headrest having a larger average HU and standard deviation compared to the moldable headrest. Overall, the HU‐derived effective density of the standard‐of‐care and 3D‐printed headrest were comparable.

#### 3.B.1 Ion chamber measurements

The results of the ion chamber measurements are presented in Table 1. The percent difference in the ionization readings between the two headrests was 0.55%. Considering the limitations of setup variation in the phantoms between the two measurements, the ionization measurements were within expected deviation. This result indicates the two headrests are dosimetrically equivalent.

**TABLE 1:** In phantom ionization chamber readings.

	3D‐printed headrest	Standard‐of‐care	Percent difference
Reading 1 (nC)	14.43	14.51	
Reading 2 (nC)	14.43	14.51	
Average	14.43	14.51	0.55%

#### 3.B.2 Film measurements

**FIG. 8:**
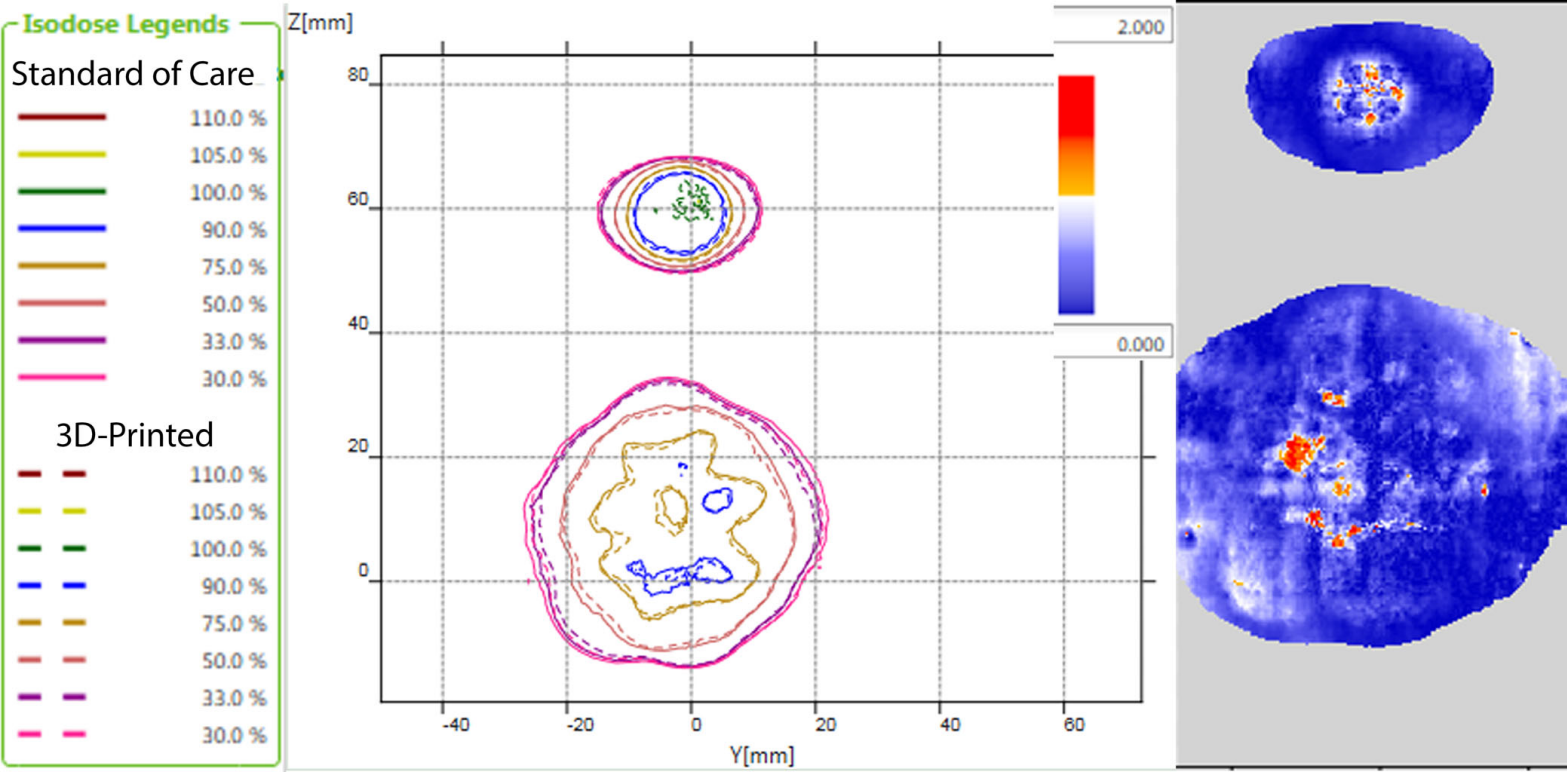
Left: isodose comparison overlay. Right: gamma index map.

The gamma index map and isodose comparison for the films are presented in Fig. 8. The calculated gamma index was 98.6% of points passing. Considering the inherent uncertainty of EBT3 film is at least 3.2%,^15^ the gamma index suggests very good agreement of the two films. These results demonstrate that the delivered dose distribution for each headrest was clinically equivalent and provides additional evidence of dosimetric equivalence of the two headrests.

### 3.C Immobilization study

**FIG. 9:**
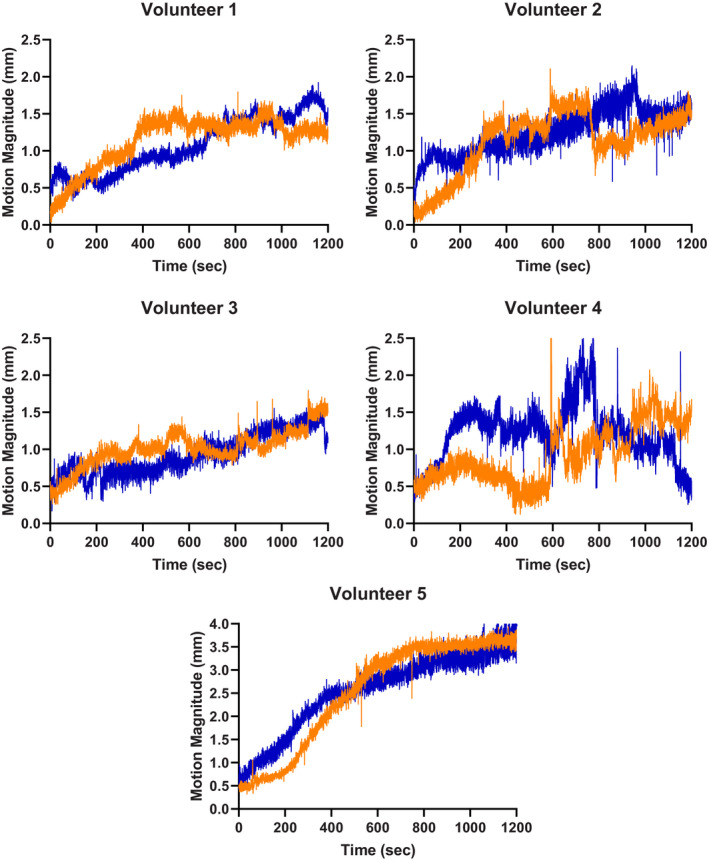
Plots of movement while immobilized in three‐dimensional‐printed headrest (blue) and standard‐of‐care headrest (orange) for each volunteer.

Plots of the vector distance vs time for each volunteer are presented in Fig. 9. Data on the mean displacement and standard deviation for each volunteer and immobilization setup are presented in Table 2. The uncertainty in the motion tracking data was estimated as 0.2 mm, which was the average vector distance recorded when tracking the ROI shown in Fig. 5 on the stationary anthropomorphic head phantom.

**TABLE 2:** Summary of VisionRT movement data recorded for volunteer immobilization study.

	Mean vector distance (mm)		Standard deviation	
	3D‐Printed	Standard‐of‐care	3D‐Printed	Standard‐of‐care
Volunteer 1	1.30	1.15	0.28	0.42
Volunteer 2	1.08	1.14	0.38	0.34
Volunteer 3	0.93	1.02	0.28	0.25
Volunteer 4	1.19	0.93	0.38	0.40
Volunteer 5	2.55	2.55	0.84	1.15
Average	1.41	1.36	0.43	0.51
*P*‐value	0.466			

Qualitatively, it can be seen in the plots in Fig. 9 that each volunteer had very similar motion trends while immobilized in the 3D‐printed headrest and the standard‐of‐care headrest. This was reflected in the recorded data, with the difference in the average motion of the volunteers for each headrest being only 0.05 mm. The paired t‐test comparing the average movement between the 3D‐printed and standard‐of‐care had a *P*‐value of 0.466, meaning there was not a statistical difference in the volunteer movement while immobilized in the two headrests. The difference in standard deviations was within 0.08 mm, with the standard deviation for the 3D‐Printed headrest being slightly smaller. These results demonstrate that the 3D‐printed headrest has comparable immobilization efficacy as a typical patient‐specific standard‐of‐care cushion. While the tape immobilization used in this study would be less effective than a full thermoplastic mask used in frameless Gamma Knife treatments, even with less restrictive head immobilization, the volunteer motion between headrests in this study was comparable and demonstrates the standardized 3D‐printed headrest would not be a limiting factor in immobilization.

## 4 DISCUSSION AND CONCLUSION

The purpose of this study was to design and validate a standardized 3D‐printed headrest for the immobilization of patients undergoing frameless Gamma Knife SRS treatment. A dosimetric study demonstrated the 3D‐printed headrest does not negatively affect delivering the intended dose to the target as planned. An immobilization study demonstrated the 3D‐printed headrest provided comparable immobilization to the current standard‐of‐care moldable headrest. Together, these results demonstrate that the 3D‐printed headrest is suitable for use in frameless Gamma Knife treatments.

This research has immediate applications for clinical use in frameless Gamma Knife treatments. As discussed in the introduction, the primary motivation for this study was the difficulty in fabricating a headrest during a patient's simulation due to time limitations, patient cooperation, and geometrical challenges. Use of the 3D‐printed standardized headrest developed in this study would effectively alleviate these challenges in generating the headrest using the current standard‐of‐care. This would not only make the simulation process more enjoyable to the patient but also shorten the time required for simulation by about 15 min, which can free up staff and machine time.

In addition to improving simulation, the 3D‐printed headrest could potentially enable patients to undergo frameless treatment who otherwise would not have been able to be treated due to collision. Targets located very anterior or inferior in the skull can be challenging to treat due to collision of the patient in the machine when positioning the target at isocenter. As shown in Fig. 7, the 3D‐printed headrest developed in this study is significantly thinner — on the order of 1 cm — than a typical moldable headrest currently used. The thinner headrest provides additional clearance, which could make the difference in being able to treat a target in a collision susceptible location. Another technique used to work around collision issues in conventional Gamma Knife treatments is to change the gamma angle, which is the angle the frame locks into the fixation mount. Typically a neutral head position, which is achieved with the 90° gamma angle, is the default angle used for frame‐based treatment. Changing the patient to be in a chin up (70° gamma angle) or chin down (110° gamma angle) head position sometimes provides the additional clearance needed to treat a target. This technique could be applied to a frameless treatment using a specially made 3D‐printed headrest. The headrest shape could be modified to guide the patient's head into a chin up or chin down position, which would mimic the effect of a gamma angle. The authors plan to further investigate this in future research.

One of the primary strengths of 3D‐printing technology is the ability to rapidly manufacture unique devices. Although this study primarily focused on validation of a standardized design, the headrest design developed in this study could be used as a foundational shape to generate patient‐specific headrests. Prior studies have demonstrated the ability to use a patient's pre‐treatment diagnostic imaging data to 3D‐print patient‐specific treatment devices.^16^, ^17^, ^18^, ^19^ The majority of Gamma Knife patients have a diagnostic MRI, which could be used to generate a patient‐specific headrest model that could be 3D‐printed prior to a simulation. This technique could offer similar time savings during simulation as using a pre‐made standardized headrest, while potentially offering better immobilization because it would be molded to the patient's specific head shape.

In conclusion, we have designed and validated a 3D‐printed headrest that can be used for patient immobilization in frameless Gamma Knife treatments. Use of the 3D‐printed headrest can alleviate the challenges associated with using the current standard‐of‐care moldable headrests. The design developed in this study can be utilized as a foundation for future research in 3D‐printed head position assisting and patient‐specific headrests.

## CONFLICT OF INTEREST

No conflict of interest.
